# Occupational Therapy

**Published:** 1917-06-02

**Authors:** 


### Occupational Therapy.

The June issue of *The Modern Hospital* (Chicago and St. Louis) will be devoted to the subject of "Occupational Therapy and Occupations for the Handicapped," a subject the importance of which has not been sufficiently realised until comparatively recent times. Among the papers to be published are "History of Occupational Therapy," by Dr. W. R. Dunton, Junr., assistant physician, Sheppard and Enoch Pratt Hospital, Towson, Md.; "The Potteries of Arequipa Sanatorium, an Experiment in the Re-education of Tuberculous Girls," by Dr. Philip King Brown, medical director of Arequipa Sanatorium, Manor, Cal.; "Remunerative Occupations for the Handicapped," by Dr. Herbert J. Hall, physician-in-charge, Devereux Mansion, Marblehead, Mass.; "Occupation Therapy in the Mental Hospital," by Dr. A. H. Ruggles, first assistant physician, Butler Hospital, Providence, R.I.; "Occupation and Diversion for Tuberculous Patients," by Dr. A. T. Laird, superintendent, Nopeming Sanatorium, Nopeming, Minn.; "Work in the Treatment of Insane Criminals," by Dr. Paul E. Bowers, medical superintendent, Indiana Hospital for Insane Criminals; "Some Principles of Occupational Therapy," by Miss Elizabeth Upham, director of art department, Milwaukee-Downer College, Milwaukee; "The Inoculation of the Bacillus of Work," by Mr. George Edward Barton, director of Consolidation House, Clifton Springs, N.Y.

